# Enzyme Activity and Lipogenesis Inhibition by Fermented Grain Using Natural Enzymes

**DOI:** 10.3390/molecules28217285

**Published:** 2023-10-26

**Authors:** Jin-Sung Jun, Ye-Lim You, Ha-Jun Byun, Kyung-Hoon Han, Jay Kim, Jea-Bum Jung, Hyeon-Son Choi, Sung-Hee Han

**Affiliations:** 1 Naraentech Co., Ltd., Sanhangni-gil, Janggun-myeon, Sejong-si 30054, Republic of Korea; ojin@naver.com; 2 Department of Food Nutrition, Sangmyung University, Hongjimun 2-Gil 20, Jongno-gu, Seoul 03016, Republic of Korea; sungheeh@korea.ac.kr (Y.-L.Y.); byunhajun@gmail.com (H.-J.B.); hsc1970@smu.ac.kr (H.-S.C.); 3 Institute of Human Behavior & Genetics, Korea University College of Medicine, Seoul 02841, Republic of Korea; hankhoon2@gmail.com (K.-H.H.); jaykim0417@hanmail.net (J.K.); 4 Wisedom Science Lab, Korea University, Seoul 02841, Republic of Korea; bluevet@korea.ac.kr

**Keywords:** grain enzyme, enzyme-rich foods, antioxidant, adipogenesis, inflammatory response, metabolic disorder

## Abstract

This study aims to compare the effects of three enzyme-rich foods, including one fermented (grain enzyme) and two non-fermented foods (enzyme foods 1 and 2), by investigating their antioxidant, anti-inflammatory, and anti-adipogenic properties. Grain enzyme exhibited the highest radical scavenging activity and was rich in antioxidant components, including total polyphenol and total flavonoid contents. Grain enzyme and enzyme foods 1 and 2 inhibited nitric oxide production by 27, 34, and 17%, respectively, at a concentration of 200 μg/mL in LPS-stimulated macrophages. Among the tested enzymes, grain enzyme demonstrated the strongest inhibition on the expression of inducible nitric oxide synthase (INOS), cyclooxygenase-2 (COX-2), and interleukin (IL)-1β, while Enzyme Food 2 exhibited the most significant suppression of IL-6 mRNA levels. Furthermore, Grain Enzyme demonstrated a stronger inhibitory effect compared to Enzyme Food 1 and 2. Grain Enzyme decreased the mRNA expression of peroxisome proliferator-activated receptor (PPAR)γ, CCAAT/enhancer-binding protein (C/EBP)α, and fatty acid-binding protein (FABP)4 by 28, 21, and 30%, respectively, at a concentration of 400 μg/mL. In summary, fermented grain enzymes outperformed non-fermented enzymes in suppressing inflammation and adipogenesis. This study highlights the anti-inflammatory and anti-adipogenic effects of grain enzyme, suggesting its potential as a valuable dietary supplement for managing metabolic disorders.

## 1. Introduction

Recent advancements in living standards have led to the westernization of modern diets, resulting in a notable increase in the consumption of animal-based foods and high-calorie instant meals [1]. As a result, the prevalence of chronic diseases such as obesity, diabetes, and hypertension has been on the rise [2]. These illnesses involve the dysregulation of inflammatory and antioxidant responses, as well as lipid metabolism [3].

Enzymes within the human body are broadly categorized into metabolic and digestive enzymes. A deficiency of these enzymes can result in metabolic disorders and impair the immune system. They are generally synthesized by cells within a healthy body or acquired through the consumption of enzyme-rich foods, including fruits, vegetables, and sprout seeds. These foods are typically cultivated under various microbial conditions.

Fermented enzyme-rich foods contain a combination of safe bioactive compounds and can serve as drug delivery systems targeting specific sites [4]. They also exert protective effects against a range of microorganisms and their metabolites in the gastrointestinal tract. Probiotic microorganisms present in fermented enzyme-rich foods ferment indigestible carbohydrates, leading to the production of simple fatty acids. These fatty acids serve as food sources for bacteria in the large intestine [5]. This process assists in restraining the growth of intestinal pathogens and regulating the secretion of appetite hormones, ultimately preventing obesity. Several studies have investigated the association between fermented foods and obesity.

One study aimed to demonstrate the positive effects of a fermented, high-protein beverage containing *Lactiplantibacillus plantarum* sub sp. on obesity. The research found that *plantarum* DK211 significantly reduced fat composition and blood lipid levels in obese rats exposed to a high-fat diet, ultimately contributing to weight management [6]. A clinical study investigated the effects of consuming cheese containing *Lactiplantibacillus plantarum* sub sp. plantarum TENSIA along with a weight-loss diet. The results revealed that obese people who consumed cheese demonstrated significant reductions in body mass index (BMI) and hypertension compared to those who did not consume cheese [7]. Another study demonstrated that providing probiotic yogurt to women with obesity and overweight problems significantly reduced total and LDL cholesterol levels, consequently leading to substantial improvements in their cholesterol profiles [8]. Furthermore, fermented rice beverages containing *Bifidobacterium* sp. strain MKK4 have been found to inhibit fat cell formation and lipogenesis, promote lipid breakdown, improve glucose-insulin anti-glycemic control, and prevent obesity [9]. 

Obesity shows a persistently low-level inflammatory state and immune cell infiltration into adipose tissue. Adipose tissue functions as an endocrine organ, storing energy in the form of lipids and secreting various factors such as leptin, adiponectin, vascular endothelial growth factor, IL-6, IL-1β, TNF-α, and complement proteins [10]. When obesity occurs, there is a shift in the phenotype of adipose tissue macrophages (ATMs) from an anti-inflammatory M2 polarization state to a pro-inflammatory M1 state. M1 macrophages exhibit increased production of pro-inflammatory cytokines and generate nitric oxide (NO) through the activation of iNOS, contributing to the chronic low-grade inflammation associated with obesity [11].

Enzyme-rich foods are primarily produced using microorganisms such as *Aspergillus* sp. and *Bacillus* sp. during the fermentation and aging processes to generate various bioactive compounds and nutrients, as well as to promote the proliferation of beneficial bacteria [12]. Consuming enzyme-rich foods can enhance the digestive processes in the human body. It reduces the amount of pancreatic and gastric juice, thus improving the growth environment for intestinal microorganisms, which contributes to intestinal health. Additionally, enzyme-rich foods can activate cellular metabolic functions, facilitating the rapid replacement of aging cells with new ones. They also possess anti-inflammatory and antibacterial properties, which help activate cells and alleviate inflammation. Moreover, they are known to enhance liver function, regulate cholesterol levels, and promote blood flow [13,14].

For these reasons, in Korea, there is growing interest in the efficacy and effectiveness of enzyme-labeled products such as leached tea and fermented beverages. Concurrently, the health supplement market is rapidly expanding owing to increasing public interest in health. However, research on enzyme products remains limited. Thus, this study focused on investigating and comparing the anti-inflammatory, antioxidant, and anti-adipogenic effects of these three enzyme-rich foods. 

## 2. Results and Discussion

### 2.1. Digestive Enzyme Activity of Enzyme-Rich Foods

**Figure 1:**
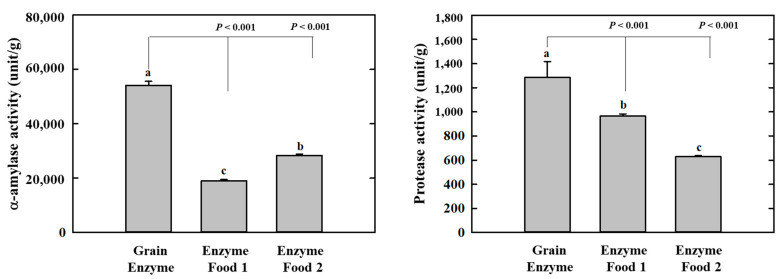
Effect of enzyme-rich foods on digestive enzyme activity. The α-amylase and protease activities of enzyme-rich foods were measured using reactions with DNS and Folin–Ciocalteu solution, respectively. The absorbances were recorded at 540 nm for α-amylase and 660 nm for protease. Values are presented as means and the standard error of the mean (mean ± S.E.M.). Different letters indicate statistically significant differences among groups (*p* < 0.05). Three replicates of each experiment were performed.

Amylases and proteases are essential digestive enzymes produced by specific microorganisms during fermentation [15,16,17,18]. The grain enzyme exhibited the highest α-amylase activity among the three enzyme-containing grain products, with an activity of 54,123.9 units/g. In comparison, enzyme foods 1 and 2 exhibited activities of 18,999.2 and 28,351.3 units/g, respectively. The α-amylase enzyme activity of the grain enzyme was 1.9–2.8 folds higher than that of enzyme food 1/2. This finding indicates that the grain-derived α-amylase present in the grain enzyme product is more abundant compared to the two competitor products. Protease activity was measured and expressed as the amount of tyrosine released from α-casein. The protease activity of the grain enzyme was higher than that of the other two products, with an activity of 1284.8 units/g. In comparison, enzyme foods 1 and 2 exhibited activities of 964.8 and 628.3 units/g, respectively (Figure 1). The protease activity of grain enzyme was 1.3 and 2.0-fold higher than that of enzyme foods 1 and 2, respectively, indicating that grain enzyme possessed superior protein-degrading capabilities in comparison to the other two products. This finding suggests that the fermented grain enzyme product is composed of ingredients with enhanced digestive enzyme activity in comparison to the non-fermented enzymes.

### 2.2. Antioxidant Activity of ‘Grain Enzymes’

Polyphenols play an important role in preventing obesity, cardiovascular disease, and cancer, which can be caused by oxidative stress [19,20]. Therefore, we determined the radical scavenging activity of each enzyme product by measuring the total polyphenol content (TPC) and total flavonoid content (TFC).

The polyphenol content, measured per 100 mg, was 3422.00, 1908.67, and 1615.33 GAE mg for grain enzyme and enzyme foods 1 and 2, respectively. The flavonoid content was 387.46, 169.17, and 146.00 CE mg for grain enzyme and enzyme foods 1 and 2, respectively (Table 1). These results indicate that both polyphenol and flavonoid contents were higher in the grain enzyme product than in enzyme foods 1 and 2. Polyphenols are known for their ability to regulate multiple signaling pathways associated with inflammatory responses, including Toll-like receptors (TLRs), nuclear factor (NF)-κB, mitogen-activated protein kinase (MAPK), phosphatidylinositol 3-kinase/protein kinase B (PI3K/AKT), and Janus kinase/signal transducer and activator of transcription (JAK/STAT), thereby suppressing the gene expression of proinflammatory cytokines [21]. Our findings indicate that the grain enzyme possesses significant potential for regulating inflammatory responses, which is attributed to its high content of total polyphenols and flavonoids among enzyme-rich foods.

**Figure 2:**
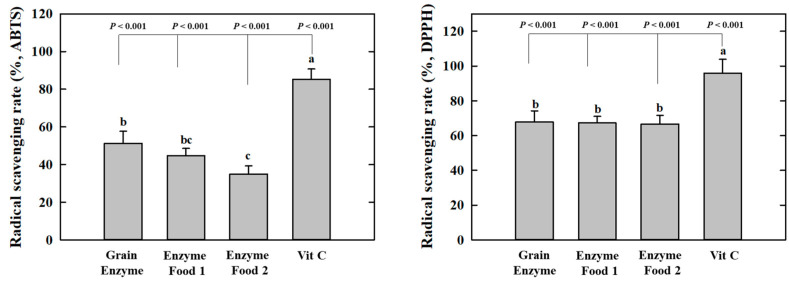
Effect of enzyme-rich foods on radical scavenging activity. Enzyme-rich foods were reacted with the ABTS or DPPH radicals, after which the absorbance was measured at 734 nm and 517 nm, respectively. Values are presented as means and the standard error of the mean (mean ± S.E.M.). Different letters indicate statistically significant differences among groups (*p* < 0.05). Three replicates of each experiment were performed.

The grain enzyme exhibited higher radical scavenging activity, as indicated by lower IC_50_ values, which represent the concentrations required to scavenge 50% of the radicals. In the context of 2,2′-Azino-bis (3-ethylbenzothiazoline-6-sulfonic acid) (ABTS), the grain enzyme and enzyme foods 1 and 2 exhibited IC_50_ values of 5.927, 9.081, and 10.56 mg/mL, respectively. In the context of 2,2-diphenyl-1-picrylhydrazyl (DPPH) and grain enzyme, enzyme foods 1 and 2 demonstrated IC_50_ values of 2.750, 3.748, and 2.583 mg/mL, respectively (Table 2, Figure 2). These results indicate that grain enzymes exhibit a higher antioxidant activity than other enzyme foods. This difference in antioxidant activity among enzyme-rich foods may potentially be attributed to differences in polyphenol and flavonoid content. Oxidative stress plays a significant role in the pathophysiology of obesity. Elevated oxidative stress promotes lipid accumulation during adipogenesis and is associated with an increased inflammatory response [22]. Thus, grain enzymes have been acknowledged for their potential to manage metabolic diseases, including obesity, through the regulation of oxidative stress. 

### 2.3. Cytotoxicity and NO Production Inhibition of Enzyme Products in Macrophages (RAW264.7)

**Figure 3:**
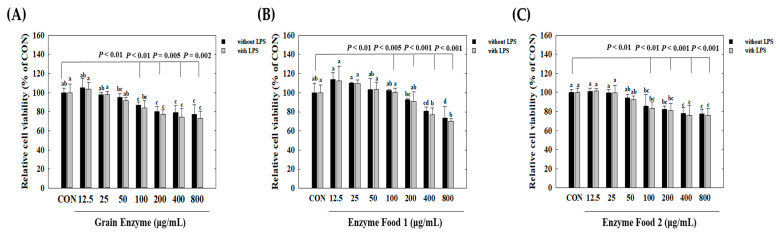
Effect of enzyme-rich foods on cell viability of RAW264.7. Raw264.7 cells were treated with enzyme-rich foods or vehicle (DMSO) for 24 h. Cell viability was examined by the MTT assay. Assays were performed in triplicate. Values are expressed as means and standard errors of the mean (means ± S.E.M.). Different letters indicate statistically significant differences versus control (CON) (*p* < 0.05). CON (control): no treatment.

We observed cell cytotoxicity in conditions inducing inflammatory responses, represented by the lipopolysaccharide (LPS)-treated group, and in normal conditions, represented by the LPS-untreated group. Initially, under conditions without LPS treatment, all three samples, grain enzyme, enzyme food 1, and enzyme food 2, showed a slight decrease (around 10%) in cell proliferation at 800 μg/mL, but no significant cell toxicity was observed (Figure 3). Under LPS-treated conditions, an increase in the concentration of samples induced cell toxicity. Grain enzyme exhibited a 23.2% reduction in cell viability at 800 μg/mL compared to the control (CON) (Figure 3A). Enzyme food 1 demonstrated cell toxicities of 18.2%, 16.3%, 22.0%, and 30.0% at concentrations of 100 μg/mL, 200 μg/mL, 400 μg/mL, and 800 μg/mL, respectively (Figure 3B). Enzyme food 2 exhibited cell toxicities of 13.5%, 13.6%, 22.0%, and 28.2% at concentrations of 100 μg/mL, 200 μg/mL, 400 μg/mL, and 800 μg/mL, respectively (Figure 3C). Therefore, to conduct experiments in concentrations with minimal or no cell toxicity, we determined the effect of samples on NO production in the range of 50 to 400 μg/mL and the effect on inflammatory mediators in the range of 50 to 200 μg/mL.

**Figure 4:**
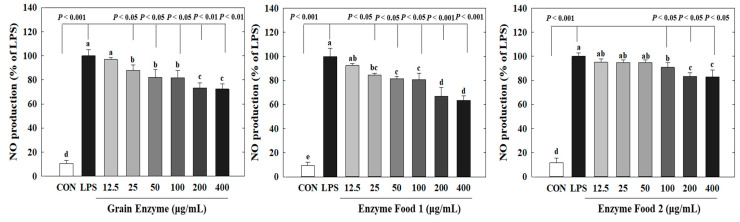
Effect of enzyme-rich foods on NO production in LPS-stimulated RAW264.7. RAW264.7 cells were treated with enzyme-rich foods or vehicle (DMSO) with or without lipopolysaccharide (LPS) for 24 h. NO production in the medium was detected by using Griess reagent. Values are presented as means and the standard error of the mean (mean ± S.E.M.). Different letters indicate statistically significant differences among groups (*p* < 0.05). CON (control): no treatment. Three replicates of each experiment were performed.

Nitric oxide (NO) plays crucial roles in neurotransmission, vascular relaxation, and cell-mediated immune responses. Specifically, upon macrophage stimulation by lipopolysaccharides (LPS), the expression of inducible NOS (iNOS) occurs, resulting in substantial production of NO [23]. Excessive NO production mediates the inflammatory response. Excessive production of inflammatory mediators can lead to an amplified immune response, exacerbating various human diseases, including diabetes, obesity, and cardiovascular diseases. We determined the effects of three enzyme-rich foods on NO production by LPS-stimulated macrophages. The RAW264.7 cell is a representative macrophage cell model to assess inflammatory responses. These cells produce nitric oxide (NO) in response to LPS, a known inducer of inflammation. Thus, LPS-induced NO production in RAW264.7 cells serves as a marker for inflammatory responses. Our study demonstrated that three samples (grain enzyme, enzyme food 1, and enzyme food 2) effectively inhibited this inflammatory response by reducing NO production in RAW264.7 cells. Grain enzyme, and enzyme foods 1 and 2 significantly inhibited NO production (Figure 4). Grain enzyme exhibited a 27% inhibition of NO production at a concentration of 200 µM, while enzyme foods 1 and 2 exhibited 34 and 17% inhibitions, respectively (*p* ≤ 0.05). 

Inducible NOS (iNOS), which is responsible for NO synthesis, increases when stimulated by inflammatory cytokines [24]. Although important for the immune response, it can adversely affect various cell types, including vascular [25] and pancreatic [26]. According to M. Perreault et al., it has been demonstrated that iNOS-derived NO may play a role in obesity-induced metabolic disorders [27].

### 2.4. Effects of Enzyme-Rich Foods on Inflammatory Gene Expression

Raw264.7 was treated with LPS to induce an inflammatory response, and then grain enzyme was treated at a concentration of 50 to 200 μg/mL to confirm inflammatory gene expression.

**Figure 5:**
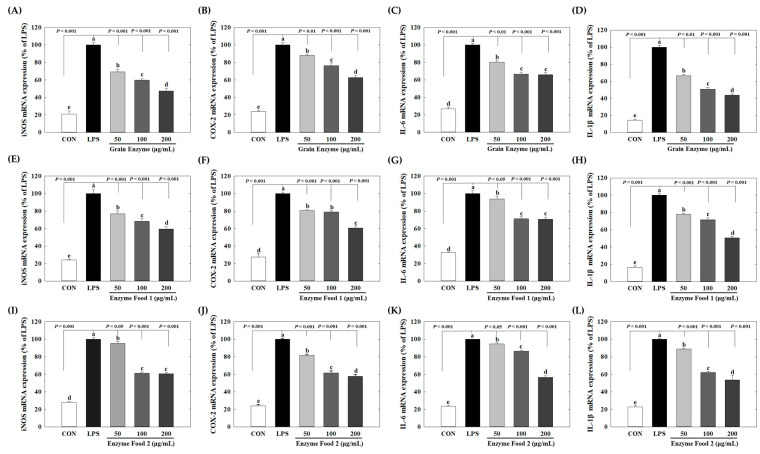
Effect of enzyme-rich foods on inflammatory mediators in RAW264.7. RAW264.7 cells were treated with enzyme-rich foods or vehicle (DMSO) with or without LPS for 24 h. Total RNA was extracted from the cells, and the mRNA expression of the indicated cytokines was analyzed by real-time PCR; expression was normalized to GAPDH. The values are presented as the mean and standard error of the mean (mean ± S.E.M.). Different letters indicate statistically significant differences among the groups (*p* < 0.05). CON (control): no treatment; LPS—only LPS treatment. Grain Enzyme: (**A**–**D**), Enzyme Food 1: (**E**–**H**), Enzyme Food 2: (**I**–**L**), iNOS: (**A**,**E**,**I**), COX-2: (**B**,**F**,**J**), IL-6: (**C**,**G**,**K**), IL-1b: (**D**,**H**,**L**).

Grain enzymes showed concentration-dependent inhibition of the mRNA levels of the inflammatory mediators iNOS and COX-2. At concentrations of 50, 100, and 200 μg/mL of grain enzyme, the mRNA expression of iNOS decreased by 30.7%, 40.1%, and 52.7%, respectively (*p* ≤ 0.05) (Figure 5A), while COX-2 mRNA expression decreased by 11.8%, 23.7%, and 37.1%, respectively (Figure 5B). The expression of pro-inflammatory cytokines was also significantly decreased. At a concentration of 200 μg/mL, grain enzyme decreased the mRNA levels of IL-6 and IL-1β by 34.0 and 56.2%, respectively (Figure 5C,D). Additionally, enzyme foods 1 and 2 inhibited the production of inflammatory mediators and cytokines. 

Enzyme foods 1 and 2 also significantly inhibited the expression of inflammatory mediators and cytokines. Enzyme food 1, at a concentration of 200 μg/mL, downregulated mRNA levels of iNOS, COX-2, IL-6, and IL-1β by 40.5, 39.3, 29.3, and 49.3%, respectively (Figure 5E–H). Similarly, enzyme food 2, at a concentration of 200 μg/mL, downregulated mRNA expression of iNOS, COX-2, IL-6, and IL-1β by 39.3, 42.2, 43.3, and 46.4%, respectively (Figure 5I–L). When 200 μg/mL concentration was used as a reference, the grain enzyme exhibited the highest activity in terms of COX-2 and IL-1β mRNA expression. However, all three enzymes effectively inhibited the expression of inflammatory mediators. It is widely acknowledged that obesity is accompanied by chronic low-grade inflammation. The adipose tissue secretes adipokines and cytokines that attract immune cells. Among these immune cells are macrophages, which, in turn, produce inflammatory cytokines, thereby inducing a persistent inflammatory response. These cytokines include IL-1β and IL-6, along with the inflammatory mediators iNOS and COX-2 [28]. Notably, iNOS and COX2 play integral roles in the inflammatory response. Specifically, iNOS is crucial for the immune response and catalyzes the production of NO from L-arginine. However, excessive iNOS expression can lead to inflammation-related diseases [29]. IL-1β and IL-6 are recognized for their involvement in chronic inflammation associated with obesity. Adipocytes and immune cells, including monocytes and macrophages, secrete IL-1β, which can damage pancreatic beta cells and lead to insulin resistance. In contrast, IL-6 contributes to the early inflammatory response by inducing protein synthesis and promoting B cell differentiation [30]. Our results show that enzyme-rich foods can inhibit the expression of inflammatory markers, suggesting their potential to control inflammatory response-mediated diseases. Specifically, grain enzyme suppresses inflammatory mediators and cytokines, along with inhibiting lipid accumulation during adipogenesis, indicating that grain enzyme has the potential to manage metabolic diseases, including obesity. 

### 2.5. Effect of Enzyme-Rich Foods on Cell Viability and Lipid Accumulation in Adipocyte

**Figure 6:**
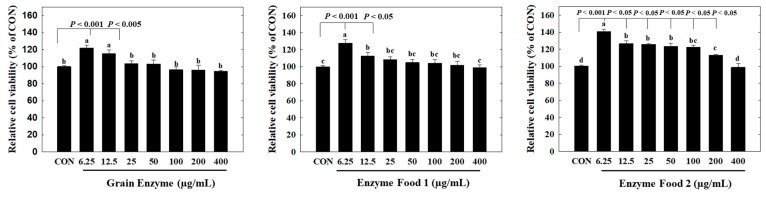
Effect of enzyme-rich foods on cytotoxicity in adipocytes. Preadipocytes or confluent 3T3-L1 cells were treated with enzyme-rich foods or vehicle (DMSO) for 48 h. Cell viability was examined by the MTT assay. Assays were performed in triplicate. Values are expressed as means and standard errors of the mean (means ± S.E.M.). Different letters indicate statistically significant differences versus control (*p* < 0.05). CON (control): no treatment.

In the cytotoxicity assay, all three products showed no observable cytotoxicity towards adipocytes at a concentration range of 400 µg/mL or lower. Interestingly, at concentrations of 6.25 and 12.5 µg/mL, we observed an increased trend in cell viability compared to the control group. In particular, Enzyme Food 1 exhibited significantly higher cell survival rates in the range of 6.25–200 µg/mL compared to the control group (Figure 6).

Overall, these results indicated that the enzyme products did not induce cytotoxicity in adipocytes and, in certain cases, even promoted cell viability at lower concentrations.

**Figure 7:**
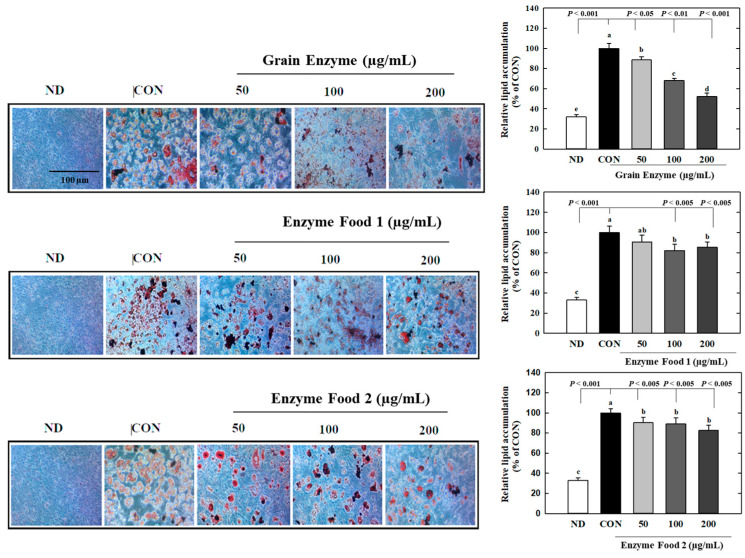
Effect of enzyme-rich foods on lipid accumulation during adipogenesis. 3T3-L1 cells were differentiated with or without enzyme-rich foods (100 μg/mL, 200 μg/mL, and 400 μg/mL) for 6 days. Lipids from each group were visualized by ORO staining and quantified by ImageJ (Version 1.53t). Values are expressed as means and standard errors of the mean (means ± S.E.M.). Different letters indicate statistically significant differences versus control (*p* < 0.05). CON (control): no treatment. ND: not differentiation. Scale bar = 100 μm.

We investigated the effects of enzyme-rich foods on lipid accumulation during adipocyte differentiation by Oil Red O (ORO) staining. The grain enzyme showed a dose-dependent inhibition of lipid accumulation, suppressing ORO-stained lipids by 12, 32, and 48% at concentrations of 50, 100, and 200 mg/mL, respectively (Figure 7) (*p* ≤ 0.05). Enzyme foods also exhibited an inhibitory effect on lipid accumulation; however, this trend was not statistically significant. This finding indicates that grain enzyme has the potential to serve as a preventive dietary supplement for addressing obesity-related health concerns.

### 2.6. Analysis of the Impact of Grain Enzyme on Adipogenic Marker Expression

We investigated the impact of three enzyme-rich foods on the expression of adipogenesis markers by isolating mRNA from preadipocyte cells, generating cDNA, and then using real-time PCR.

**Figure 8:**
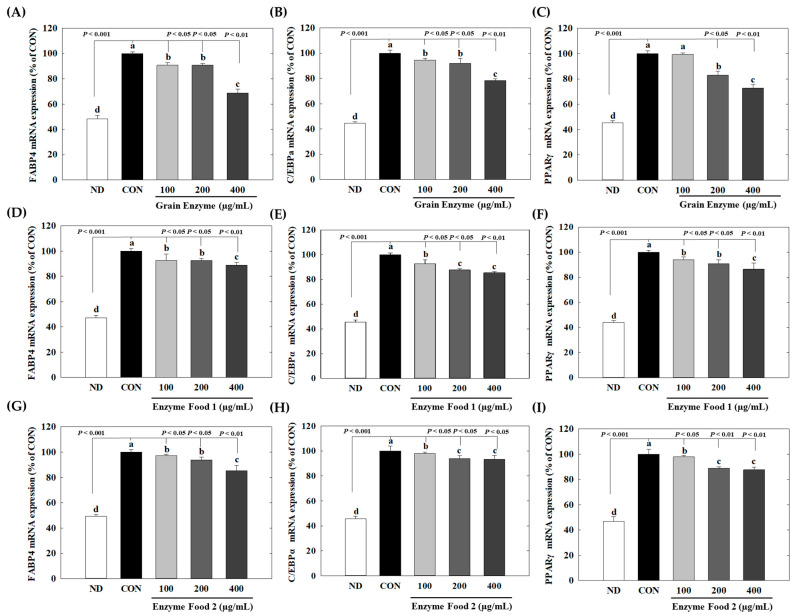
Effect of enzyme-rich foods on adipogenic marker expression. 3T3-L1 cells were differentiated with or without enzyme-rich foods (100 μg/mL, 200 μg/mL, and 400 μg/mL) for 6 days. Total RNA was extracted from cells, and the levels of mRNA expression of PPARγ, C/EBPα, and FABP4 were assessed by real-time RT-PCR and normalized to GAPDH. Values are expressed as means and standard errors of the mean (means ± S.E.M.). Different letters indicate statistically significant differences versus control (*p* < 0.05). CON (control): no treatment. ND: not differentiation. Grain Enzyme: (**A**–**C**), Enzyme Food 1: (**D**–**F**), Enzyme Food 2: (**G**–**I**), FABP4: (**A**,**D**,**G**), C/EBPα: (**B**,**E**,**H**), PPARγ: (**C**,**F**,**I**).

At a concentration of 400 µg/mL, grain enzyme exhibited significant inhibitory effects on the expression of peroxisome proliferator-activated receptor gamma (PPARγ) (28%), CCAAT/enhancer-binding protein alpha (C/EBPα) (21%), and fatty acid-binding protein 4 (FABP4) (30%) genes (*p* ≤ 0.05). On the other hand, Enzyme Food 1 showed inhibitory effects of 14–15% on PPAR and C/EBPα and 11% on FABP4. Enzyme Food 2 demonstrated inhibition effects of 16, 6, and 18% for PPARγ, C/EBPα, and FABP4, respectively (Figure 8). These findings indicated that, among the three enzyme products, the grain enzyme showed the most potent inhibitory effect on adipogenic markers.

Adipose-tissue development is intricately associated with adipocyte differentiation [31]. This process involves the expression of adipogenic transcription factors and the synthesis of lipid formation-related proteins [32]. Therefore, targeting the inhibition of adipogenesis and lipogenesis emerges as a central approach to tackling obesity. C/EBPα, PPARγ, and SREBP-1c are known to influence adipogenesis [33]. Increased expression of C/EBPα and PPARγ is the primary requirement for a preadipocyte to mature into an adipocyte during adipogenesis. FABP4, a downstream target of both C/EBPα and PPARγ, plays an important role in lipid accumulation in adipocytes. SREBP1c serves as a transcription factor that regulates fatty acid synthesis by promoting the gene expression of fatty acid synthase (FAS) [34,35,36]. In our data, the grain enzyme showed a superior inhibitory effect on the expression of adipogenic factors compared to other enzyme-rich foods. This suggests that the grain enzyme exhibits a more significant inhibition of lipid accumulation by suppressing adipogenesis.

## 3. Materials and Methods

### 3.1. Samples

Samples were grain enzyme, enzyme food 1, and enzyme food 2. The grain enzyme sample is a fermented grain food. The black rice, Job’s tear, and brown rice were fermented by *Aspergillus oryzae*. It was fermented, dried, and granulated. The samples of enzyme foods 1 and 2 were not fermented. It was a commercial product with α-amylase and protease.

### 3.2. Measurement of α-Amylase Activity

The α-amylase activities of the samples were measured using the DNS method. Each enzyme powder (0.5 g) was mixed with 4 mL of 0.1 N sodium dihydrogen phosphate buffer solution (pH 7.0, Sigma-Aldrich Co., St. Louis, MO, USA) and agitated for 40 min. After agitation, the mixture was centrifuged at 3000 rpm for 10 min (1236MG, GYROZEN Co., Ltd., Daejeon, Republic of Korea) to obtain the supernatant. From the supernatant, 0.5 mL was aspirated and mixed with 2.5 mL of 1% starch solution, 6.5 mL of sodium dihydrogen phosphate buffer solution, and 0.5 mL of 0.1% calcium chloride solution. The mixture was then incubated at 37 °C for 20 min. Subsequently, the reaction was stopped by heating at 100 °C for 10 min, followed by cooling at 4 °C and centrifugation at 10,000 rpm for 10 min to obtain the supernatant. To the reaction mixture (0.4 mL), 1.2 mL of DNS solution was added, and the absorbance was measured at 540 nm using a spectrophotometer (Ultraspec-2100pro, Amersham Co., Uppsala, Sweden). In control experiments, water or a heated test solution were used instead of the supernatant. Additionally, glucose was diluted to concentrations ranging from 10 mg to 1000 mg, and the absorbance was measured after the reaction to construct a standard curve.

### 3.3. Measurement of Protease Activity

The protease activity was measured using the modified Kunitz method [37]. In a 0.2 M sodium phosphate buffer (pH 7.0), 0.5 mL of the sample solution was obtained by mixing 0.5 g of enzyme powder with 4 mL of buffer and shaking for 40 min, followed by centrifugation. Subsequently, 0.5 mL of 0.6% casein solution (*w/v*, Duksan Pure Chemicals Co., Ltd., Ansan, Republic of Korea) was added, and the mixture was incubated at 37 °C and 100 rpm shaking in a water bath (BS-31, Jeio Tech Co., Ltd., Seoul, Republic of Korea) for 20 min. After incubation, 2.0 mL of 0.44 M trichloroacetic acid (Acros Organics, Newark, NJ, USA) was added, and the mixture was centrifuged at 3000 rpm for 10 min (1236MG, GYROZEN Co., Ltd., Daejeon, Republic of Korea). To 1.5 mL of the supernatant, 1.0 mL of 0.55 M sodium carbonate anhydrous (Duksan Pure Chemicals Co., Ltd., Ansan, Republic of Korea) and 1.0 mL of 1 N Folin ciocalteu solution (Sigma-Aldrich Co., St. Louis, MO, USA) were added. The mixture was then incubated at 37 °C for 30 min for color development. Absorbance was measured at 660 nm using a spectrophotometer (Ultraspec-2100pro, Amersham Co., Uppsala, Sweden). Protease activity was calculated based on a standard curve prepared using quantified tyrosine (Sigma-Aldrich Co., St. Louis, MO, USA) at concentrations of 0, 12.5, 25, 50, 100, and 200 μg/mL.

### 3.4. Total Phenol and Flavonoid Content

The total phenolic content of each sample was determined using the method described by Velioglu et al. [38]. Briefly, the samples were dissolved in distilled water to specific concentrations. Then, a 2% Na_2_CO_3_ solution (2 mL) was added, and the mixture was allowed to stand for 3 min. Subsequently, 100 μL of a 50% Folin–Ciocalteu reagent was added. After a 30 min reaction, the absorbance was measured at 750 nm. The results were expressed as mg gallic acid equivalent (GAE) per gram of sample, based on a standard calibration curve using gallic acid as the reference substance. The total flavonoid content was determined using a modified version of the method described by Jia et al. [39]. Distilled water (1.25 mL) was added to each sample, followed by the addition of 75 μL of 5% NaNO_2_ solution. The mixture was allowed to stand for 5 min. Following this, 150 μL of 10% AICl_3_·6H_2_O solution was added, and the mixture was allowed to stand for an additional 6 min. To the reaction mixture, 500 μL of 1M NaOH and 275 μL of distilled water were added, and the absorbance was measured at 510 nm. A standard calibration curve was prepared using catechin as the reference substance, and the total flavonoid content of the sample was expressed as mg of catechin equivalent (CE) per gram.

### 3.5. Measurement of ABTS and DPPH Radical Scavenging Activity

ABTS radical (ABTS+) was generated by mixing a solution of 7 mM ABTS and 2.45 mM potassium persulfate and allowing it to react at 25 °C in the dark for 24 h. The resulting solution was then diluted with 100% ethanol to obtain an ABTS radical solution with an absorbance value of 0.7 at 734 nm. For the measurement of DPPH radical scavenging activity, a solution of 200 μM DPPH in methanol was prepared and diluted with methanol to obtain an absorbance value of 0.7 at 517 nm. The samples were diluted to various concentrations, mixed with a diluted DPPH solution, and allowed to react in the dark for 30 min. Absorbance was then measured at 517 nm. A radical scavenging curve was plotted for each concentration, and the concentration at which 50% of the radicals were scavenged (IC50) was determined by comparison with the control group using catechin. The radical scavenging activity was calculated using the following equation:

ABTS or DPPH radical scavenging activity (%) = (1 − sample absorbance) × 100

### 3.6. Cell Viability

To evaluate the cytotoxicity of the samples on RAW 264.7, or 3T3-L1 cells, the cells were seeded in a 96-well plate at a concentration of 1 × 10^4^ and 1 × 10^5^ cells/well. After incubating the cells for 24 h (RAW264.7) or 48 h (3T3-L1), the samples and/or LPS (1 mg/mL) were added at the indicated concentrations. After 24 h of incubation, the culture medium was carefully removed, and the cells were treated with a diluted solution of MTT reagent (0.5 mg/mL) in serum-free Dulbecco’s modified eagle medium (DMEM) medium. The cells were then incubated at 37 °C for 45 to 60 min. MTT formazan was dissolved in dimethyl sulfoxide (DMSO), and absorbance was measured at 550 nm to evaluate cell toxicity.

### 3.7. Cell Culture and Differentiation

The mouse-derived preadipocytes (3T3-L1 preadipocytes), obtained from the American Type Culture Collection (CL-173: ATCC, Manassas, VA, USA), were cultured in DMEM medium containing 10% bovine serum (BS), 1% penicillin-streptomycin, 584 mg/L L-glutamine, and 110 mg/L sodium pyruvate. The medium was changed every 2 days, and the cells were passaged using a PBS solution and 0.05% trypsin-EDTA. The cells were evenly seeded in a 12-well plate, and after reaching confluency, which typically took 2–3 days, differentiation was induced by treating the cells with a differentiation cocktail consisting of 10% fetal bovine serum (FBS), 1% penicillin-streptomycin, and DMEM supplemented with 1 μM dexamethasone (DEX), 0.5 mM IBMX, and 1.67 μM insulin. The differentiation process was performed for six days. Starting on the second day of differentiation, the medium was replaced with DMEM containing 1.67 μM insulin and 10% FBS for two days. Subsequently, the medium was replaced every two days with DMEM supplemented with 10% FBS. After eight to 10 days, the extent of adipocyte differentiation was observed under a microscope. For the macrophage cell culture and stimulation, Raw264.7 cells, obtained from Korean Cell Line Bank (KCLB: Seoul, Republic of Korea), were cultured in DMEM supplemented with 1% PS, 10% FBS, and 1.5 g/L sodium bicarbonate. Macrophages were seeded at a density of 1 × 10^5^ cells/well in a six-well plate. The cells were treated either with enzyme-rich food extracts or LPS at a concentration of 1 mg/mL when they reached 80% confluence. The sample was pre-treated for 2 h before LPS stimulation to assess its anti-inflammatory effect. Both cell lines were maintained in a CO_2_ incubator at 37 °C with 5% CO_2_.

### 3.8. Oil Red O Staining

Enzyme-rich food extracts were added at various concentrations (50, 100, and 200 μg/mL) and cultured for six days. After removing the medium, the cells were fixed in 10% formalin at 25 °C for 5 min. After washing with distilled water, an Oil Red O solution was added, and lipid staining was performed for 24 h. Following staining, the cells were rinsed with distilled water, and the accumulation of lipid droplets within the cells was observed using a microscope equipped with a camera (Leica DM 2500; Wetzlar, Germany). The effect of enzyme-rich foods on adipogenic lipid accumulation was observed through photography. Stained fat was quantified using the ImageJ software (Version 1.53t) (NIH, Bethesda, MD, USA).

### 3.9. Real-Time RT-PCR

Total RNA was extracted from RAW264.7 or 3T3-L1 cells using TRIzol reagent (Invitrogen, Carlsbad, CA, USA) according to the manufacturer’s protocol. The RNA content in each group was quantified using an INNO-M microspectrophotometer (Seongnam-si, Republic of Korea). One microgram of RNA was used to synthesize cDNA using a LaboPass cDNA synthesis kit (Cosmo Genetech Co., Ltd., Seoul, Republic of Korea). The PCR reaction mixture comprised cDNA, targeted primers (Table 1), and SYBR Green PCR Master Mix (Applied Biosystems, Waltham, MA, USA). Specific genes (iNOS, COX-2, IL-1β, and IL-6) were amplified for each primer pair using an AriaMx Real-Time PCR system (Agilent Technologies, Santa Clara, CA, USA).

### 3.10. Statistical Analysis

All experimental results were subjected to statistical analysis using the SPSS (Statistical Package for the Social Sciences, SPSS Inc., Chicago, IL, USA) software (Version 24.0). The results of all measurements are presented as means ± standard deviation. Significance analysis was conducted using a one-way ANOVA test, and significance was determined at the *p* < 0.05 level using Duncan’s multiple-range test. All letters were in comparison to the control group.

## 4. Conclusions

In conclusion, we examined the digestive enzyme activities of enzyme-rich foods and investigated their antioxidant effects. Among these enzymes, the grain enzyme, a grain-fermented enzyme, exhibited the highest digestive enzyme activity and antioxidant effects. Subsequently, we explored the effects of enzyme-rich foods on inflammatory responses. We found that grain enzymes effectively suppressed NO production and gene expression of inflammatory mediators induced by LPS treatment in macrophages. In 3T3-L1 adipocytes, grain enzymes significantly inhibited lipid accumulation during adipogenesis by regulating adipogenic transcription factors. This study confirmed the significant antioxidant, anti-inflammatory, and anti-adipogenic effects of grain-fermented enzymes, underscoring their potential as preventive dietary supplements for managing obesity and inflammatory responses.

## Figures and Tables

**Table 1:** Total polyphenol and flavonoid content.

	Samples		
	Grain Enzyme (100 mg)	Enzyme Food 1 (100 mg)	Enzyme Food 2 (100 mg)
TPC (GAE mg)	3422.00 ± 352.1 ^a^	1908.67 ± 89.3 ^b^	1615.33 ± 15.9 ^c^
TFC (CE mg)	387.46 ± 31.2 ^a^	169.17 ± 13.8 ^b^	146.00 ± 12.8 ^b^

TPC—Total Polyphenol Content; TFC—Total Flavonoid Content. Values are presented as means and standard error of the mean (mean ± S.E.M.). Different letters indicate statistically significant differences among groups (*p* < 0.05). Three replicates of each experiment were performed.

**Table 2:** Translation of ABTS and DPPH scavenging activities.

	Samples			
	Grain Enzyme (100 mg)	Enzyme Food 1 (100 mg)	Enzyme Food 2 (100 mg)	Vitamin C
ABTS (IC_50_, mg/mL)	5.927 ± 0.32 ^c^	9.081 ± 0.21 ^b^	10.56 ± 0.53 ^a^	0.06 ± 0.01 ^c^
DPPH (IC_50_, mg/mL)	2.750 ± 0.41 ^b^	3.748 ± 0.18 ^a^	2.583 ± 0.17 ^b^	0.03 ± 0.01 ^c^

ABTS—2,2′-Azino-bis (3-ethylbenzothiazoline-6-sulfonic acid); DPPH—2,2-diphenyl-1-picrylhydrazyl. Values are presented as means and standard error of the mean (mean ± S.E.M.). Different letters indicate statistically significant differences among groups (*p* < 0.05). Three replicates of each experiment were performed.

## Data Availability

All data generated or analyzed during this study are included in this published article.
